# Syphilis, Gonorrhea, and Chlamydia Notifications in Galați County, Romania (2017–2025): A Retrospective Surveillance Analysis

**DOI:** 10.3390/diseases14100373

**Published:** 2026-10-11

**Authors:** Ionelia State, Laura Cristescu-Budală, Rares-Nicolae Vadana, Alin-Laurentiu Tatu, Laurentiu-Tony Hangan, Simona-Dana Mitincu-Caramfil

**Affiliations:** 1 Faculty of Medicine, “Ovidius” University of Constanța, 900470 Constanța, Romaniatony@medcon.ro (L.-T.H.); 2 Galați County Public Health Directorate (Direcția de Sănătate Publică Galați), 800010 Galați, Romania; 3 Faculty of Medicine and Pharmacy, Research Centre in the Medical-Pharmaceutical Field, “Dunarea de Jos” University of Galați, 800008 Galați, Romania; simona.mitincu@ugal.ro; 4 Multidisciplinary Integrated Centre of Dermatological Interface Research (MICDIR), “Dunarea de Jos” University of Galați, 800385 Galați, Romania; dralin_tatu@yahoo.com; 5 Department of Dermatology, “Saint Parascheva” Infectious Disease Clinical Hospital, 800179 Galați, Romania; 6 Clinical Medical Department, Faculty of Medicine and Pharmacy, “Dunarea de Jos” University of Galați, 800385 Galați, Romania

**Keywords:** syphilis, sexually transmitted infections, epidemiological surveillance, Romania, case detection

## Abstract

Background/Objectives: Sexually transmitted infections (STIs) are resurgent globally, yet sub-national epidemiological data from Romania remain sparse. This study characterizes long-term STI trends in Galați County (population ~500,000) and examines whether observed morbidity patterns reflect transmission dynamics or surveillance system performance. Methods: Retrospective analysis of all notified STI cases in Galați County, 2017–2025 (nine years; *n* = 253), from the County Public Health Directorate surveillance database. Data included demographics, clinical stage, detection route, and temporal trends. Quasi-Poisson regression with a population offset estimated the annual change in notification rates; standardized morbidity ratios (SMR) compared local to national levels (2021–2024). Results: Over nine years, 253 cases were notified: 204 syphilis (80.6%), 49 gonorrhea (19.4%), and no chlamydia. Notification rates declined across 2017–2025 (rate ratio per year = 0.87; 95% CI 0.78–0.97; *p* = 0.014), reached a trough in 2020 and recovered partially thereafter, a pattern that may reflect variation in case detection, although testing volumes were not available to confirm this. Over 2021–2024, syphilis notification rates exceeded national levels in both males (SMR = 2.35; 95% CI 1.79–3.03; *p* < 0.0001) and females (SMR = 1.76; 95% CI 1.15–2.57; *p* = 0.011). Primary-stage disease was recorded in 13 of 204 syphilis cases (6.4%); antenatal screening identified 32 maternal infections over nine years with zero congenital syphilis notifications. Conclusions: The notification pattern is compatible with an important role of case detection, in particular the contrast between structured antenatal screening and opportunistic detection pathways, but testing and outcome data are needed to confirm this interpretation.

## 1. Introduction

Sexually transmitted infections (STIs) remain a significant global public health challenge, with the World Health Organization estimating that more than one million curable STIs are acquired daily worldwide [1]. After a prolonged period of decline throughout the latter half of the twentieth century, syphilis experienced a marked resurgence beginning in the early 2000s across high-income nations, driven by evolving patterns of sexual contact, erosion of population-level screening programs, and delays in clinical diagnosis and treatment [2,3]. This reversal has been consistently documented in the United States, Western Europe, and increasingly in Central and Eastern Europe, where surveillance systems have registered climbing notification rates that reflect both true epidemiological changes and intensified case detection [4].

At the European Union and European Economic Area (EU/EEA) level, syphilis notifications have traced an upward trajectory over the past decade, with a pronounced male predominance and clustering among men who have sex with men (MSM) in most reporting countries [4]. Romania has maintained syphilis notification rates below the EU/EEA mean, yet data from the National Institute of Public Health (INSP) document a pattern broadly aligned with European trends, including recent increases and subsequent stabilization [5]. The Romanian epidemiological profile does, however, diverge from the European pattern in one critical respect: heterosexually transmitted syphilis cases outnumber reports from MSM populations, and notifications of gonorrhea and Chlamydia trachomatis remain persistently low, a pattern more plausibly explained by limited testing and under-detection than by genuinely low transmission, although national testing data to confirm this are not available. Historical surveillance data spanning three decades support this interpretation, showing chlamydia notifications chronically suppressed relative to syphilis and gonorrhea, a discrepancy attributed to inadequate testing infrastructure rather than biological absence [6].

Despite syphilis’ recognized importance as a surveillance priority, sub-national epidemiological investigations remain scarce in the recent Romanian literature. National aggregate data may obscure substantial variation in local case burden and detection capacity between counties. Galați County, located in south-eastern Romania, comprises approximately 500,000 inhabitants distributed across mixed urban and rural areas. To our knowledge, no published analysis of the STI notification pattern in this county exists in the peer-reviewed literature, creating a gap in evidence available to local public health planners and clinicians.

This nine-year retrospective study examines all officially notified STI cases in Galați County between 2017 and 2025, with two principal aims: first, to characterize the epidemiological pattern across the full study period, documenting temporal trends, sex distribution, clinical stage at diagnosis, and detection routes; second, to explore whether the observed distribution and temporal dynamics are more consistent with changes in transmission or with the structure and variable intensity of case detection and reporting. Notification data record only infections that were diagnosed and reported; they do not capture the number of infections, the number of persons tested, or test positivity. This second aim is therefore exploratory: the study cannot separate these explanations definitively, but it can assess which of them is more compatible with the internal patterns of the data. We expected that structured screening circuits, notably mandatory antenatal syphilis screening, would contribute a consistent share of detected cases, whereas opportunistic pathways would fluctuate with changes in clinical activity and testing capacity. Chlamydia trachomatis, although notifiable, is considered in this framework as a marker of detection capacity rather than as an outcome whose true frequency can be estimated. The nine-year horizon allows assessment of sustained trends across the COVID-19 (coronavirus disease) pandemic disruption, which created a natural experiment in case-finding variation.

## 2. Materials and Methods

### 2.1. Study Design and Setting

This was a retrospective, observational, descriptive, and analytical study of sexually transmitted infection (STI) cases officially notified in Galați County, south-eastern Romania, between 2017 and 2025. Galați County had a mixed urban–rural population that declined from approximately 511,000 in 2018 to 493,000 in 2024 [7], of which just over half resided in urban areas at the time of the 2021 national census [8]. The analysis was restricted to the three STIs subject to case notification under Ministry of Health Order No. 1342/2013: syphilis, gonorrhea, and *Chlamydia trachomatis* infection. HIV (Human Immunodeficiency Virus)infection is reported through a separate national surveillance program and was not a study outcome; in the county STI database, HIV was recorded only as a co-testing variable attached to each notified case. Other STIs (e.g., trichomoniasis, genital herpes, human *papillomavirus* infection, *Mycoplasma genitalium*) are not notified as cases in this system; where tested, herpes simplex virus and *Mycoplasma genitalium* were recorded only as co-infections of notified syphilis or gonorrhea cases (Section 3.6).

### 2.2. Data Sources

Case-level data were obtained from the surveillance database of the Galați County Public Health Directorate (DSP Galați), the decentralized territorial authority responsible for notifiable-disease surveillance under the Romanian Ministry of Health and were released by the Directorate in de-identified form (see Section 2.5). Each record corresponded to a single notified case and included, in addition to etiology, variables on age, sex, area of residence (urban/rural), clinical stage at diagnosis, reason for detection, the healthcare unit where the case was identified, HIV co-testing status, and, where recorded, partner-notification outcomes. For each notified case, the form also records whether testing for other sexually transmitted and blood-borne infections (HIV, *Chlamydia trachomatis*, hepatitis B and C, herpes simplex virus and *Mycoplasma genitalium*) was performed at the time of diagnosis and its result. Testing for *Chlamydia trachomatis* was not part of a systematic screening program. According to the dermatovenereology service contributing most notifications, patients with syphilis, and particularly those with gonococcal urethritis, are frequently tested for chlamydia and for other sexually transmitted and blood-borne infections, but these results are not systematically recorded on the notification form. The database contains no information on chlamydia tests performed in persons who were not notified with syphilis or gonorrhea. Records contained no direct identifiers, and all results are reported in aggregated form. The unit of analysis was the notification: each record corresponds to one notified episode of infection. Repeat infections diagnosed in the same person in separate episodes were notified and are therefore counted as separate cases; because records were de-identified, notifications belonging to the same person could not be linked. Duplicate records were sought using the unique case code assigned by DSP Galați, and no duplicate codes were found among the 240 coded records; the 13 records without a code (2017–2018) were checked individually against age, sex, residence area, etiology, and detection date. The study included cases notified by DSP Galați among residents of Galați County; the place of infection was recorded as Galați County for 228 cases, another Romanian county for 8, and another country for 17. Cases were assigned to calendar years according to the annual surveillance files, which are organized by year of notification; the date of detection fell in the same calendar year for 248 of the 253 records. Completeness was assessed by comparing annual totals with aggregate county reports for 2021–2025 and by tabulating missing values for each variable analyzed. As an internal data-quality check, case totals and the syphilis/gonorrhea breakdown for 2021–2025 extracted from the case-level database were compared against previously reported aggregate surveillance figures for the same period and found to be identical. Sex-specific national syphilis notification rates for 2021–2024, used as the reference for indirect standardization, were obtained from the annual surveillance reports of the National Institute of Public Health and its National Centre for Surveillance and Control of Communicable Diseases (INSP–CNSCBT) [5]; these national figures correspond to those reported by Romania to ECDC (European Centre for Disease Prevention and Control) [9,10]. The rates applied for each year are given in Table S1.

### 2.3. Case Definitions and Legal Framework

Case definitions, clinical staging and notification procedures followed the methodology approved by Romanian Ministry of Health Order No. 1342/2013 on the surveillance of sexually transmitted infections [11]. Personal data were processed in accordance with Regulation (EU) 2016/679 (General Data Protection Regulation) [12]. Laboratory confirmation of syphilis required demonstration of *Treponema pallidum* in lesion exudates or tissues by dark-field microscopy, direct immunofluorescence or PCR(Polymerase Chain Reaction), or a reactive non-treponemal screening test (VDRL - Venereal Disease Research Laboratory, RPR- Rapid Plasma Reagin) confirmed by a treponemal test (TPHA - Treponema Pallidum Hemagglutination Assay, EIA - Enzyme Immunoassay, FTA-abs - fluorescent treponemal antibody absorption or immunoblot) or by detection of treponemal IgM (Immunoglobulin M) antibodies; the tests performed are recorded for each case. Stage was assigned by the diagnosing dermatovenereologist as primary, secondary, early latent (laboratory criteria without clinical manifestations, with a compatible history or documented seroconversion within the previous 12 months), late latent (more than 1 year), latent of unspecified duration, or tertiary. Gonorrhea required isolation of *Neisseria gonorrhoeae* by culture, detection by nucleic acid amplification or another test, or identification in a urethral smear in men by direct microscopy. The same case definitions were applied throughout 2017–2025. Congenital syphilis was defined according to the same order [11]: a confirmed case is an infant or child under 2 years of age with *Treponema pallidum* demonstrated by dark-field microscopy or direct immunofluorescence in the umbilical cord, placenta, nasal secretions or mucocutaneous lesions, with *T. pallidum*-specific IgM antibodies (FTA-abs, EIA), or with a reactive non-treponemal test and a non-treponemal (VDRL) titer at least fourfold higher than the maternal titer; a probable case is a child under 2 years with compatible clinical signs and either an epidemiological link to a mother with untreated or inadequately treated syphilis during pregnancy, or a reactive VDRL test in cerebrospinal fluid or reactive treponemal and non-treponemal tests in maternal serum. Congenital cases are diagnosed and confirmed by a county commission comprising a neonatologist, the county coordinating dermatovenereologist, and the county epidemiologist, and are notified on a dedicated form, separate from the STI notification form used in this study. Under the same order, serological testing for syphilis is mandatory for all pregnant women at the first antenatal visit and in the seventh month of pregnancy, and a non-treponemal test is performed in all parturients within the 2 days before or the first day after delivery; county public health directorates provide free testing of pregnant women by quantitative VDRL and TPHA, and infants of seroreactive mothers are evaluated with quantitative non-treponemal serology.

### 2.4. Statistical Analysis

Descriptive statistics comprised absolute and relative frequencies, with exact Clopper–Pearson 95% confidence intervals for key proportions. The secular trend in annual notification rates was assessed using Poisson regression with centered calendar year as the explanatory variable and the logarithm of the resident county population as an offset; the exponentiated coefficient was interpreted as the annual rate ratio (RR). Because annual counts were overdispersed (Pearson dispersion statistic 4.9), standard errors were scaled by the Pearson dispersion (quasi-Poisson model). A negative binomial model and a model excluding 2020 were fitted as sensitivity analyses. Sex-specific standardized morbidity ratios (SMR) were computed via indirect standardization against national reference rates for the 2021–2024 sub-period, for which directly comparable national data were available. For each sex, the expected number of syphilis cases was calculated by multiplying the national sex-specific syphilis notification rate for each year from 2021 to 2024 by the corresponding male or female usually resident population of Galați County on 1 January (Eurostat [13], the same denominators as in Table 1) and summing over the four years; the national rates, county populations and expected counts are given in Table S1. Exact 95% confidence intervals for the SMR were derived from the Poisson distribution of the observed count (chi-square method), and two-sided *p*-values were obtained from an exact Poisson test of the observed against the expected count (scipy.stats.poisson); the analysis code is available from the corresponding author on request. Because Galați County notifications are included in the national totals published by INSP–CNSCBT, the study population is nested within the national reference population, and national figures excluding the county were not available. Galați County accounted for 2.6% of the Romanian population in each year from 2021 to 2024 (Eurostat, demo_r_pjanaggr3 [13]); including the county in the reference moves the national rate slightly towards the county rate and therefore biases the SMR towards 1, so that the nested SMRs of 2.35 in men and 1.76 in women correspond to approximately 2.44 and 1.80 against the rest of the country. The reported excesses are thus slightly conservative. Between-group comparisons used Fisher’s exact test, and the goodness of fit of the clinical-stage distribution was assessed with a chi-square test. All analyses were performed in Python 3 (scipy.stats, statsmodels), with a two-sided significance threshold of α = 0.05. Unless otherwise specified, analyses draw on the full 2017–2025 dataset.

### 2.5. Ethics

Data were obtained through a formal request submitted under Law No. 544/2001 on free access to public-interest information and were provided by DSP Galați in de-identified, case-level form [14]. DSP Galați does not operate a dedicated research ethics committee. Given the retrospective, non-interventional design, the exclusive use of already-collected, de-identified administrative surveillance data, and the absence of any direct contact with the individuals concerned, formal ethical review was not required, consistent with the provisions of Regulation (EU) 2016/679 (GDPR) on the processing of de-identified data for research purposes [12]. All results are reported in aggregated form to prevent re-identification.

## 3. Results

### 3.1. Overall Distribution by Etiology

Between 2017 and 2025, 253 sexually transmitted infection (STI) cases were officially notified in Galați County. Syphilis accounted for 204 cases (80.6%) and gonorrhea for 49 (19.4%); no case of *Chlamydia trachomatis* infection was recorded in any of the nine years, as shown in Table 1. *Chlamydia* test results were documented on the notification form for only 8 of the 253 notified cases (3.2%; 7 syphilis and 1 gonorrhea case, all notified in 2020–2025), all negative (Section 3.6). Because testing is performed more often in clinical practice than it is recorded, this figure reflects the documentation of testing in the surveillance system rather than the frequency of testing, and the absence of chlamydia notifications cannot be interpreted as an absence of infection. This etiological profile contrasts sharply with the pattern reported across the EU/EEA, where chlamydia is consistently the most frequently notified bacterial STI [15].

**Figure 1:**
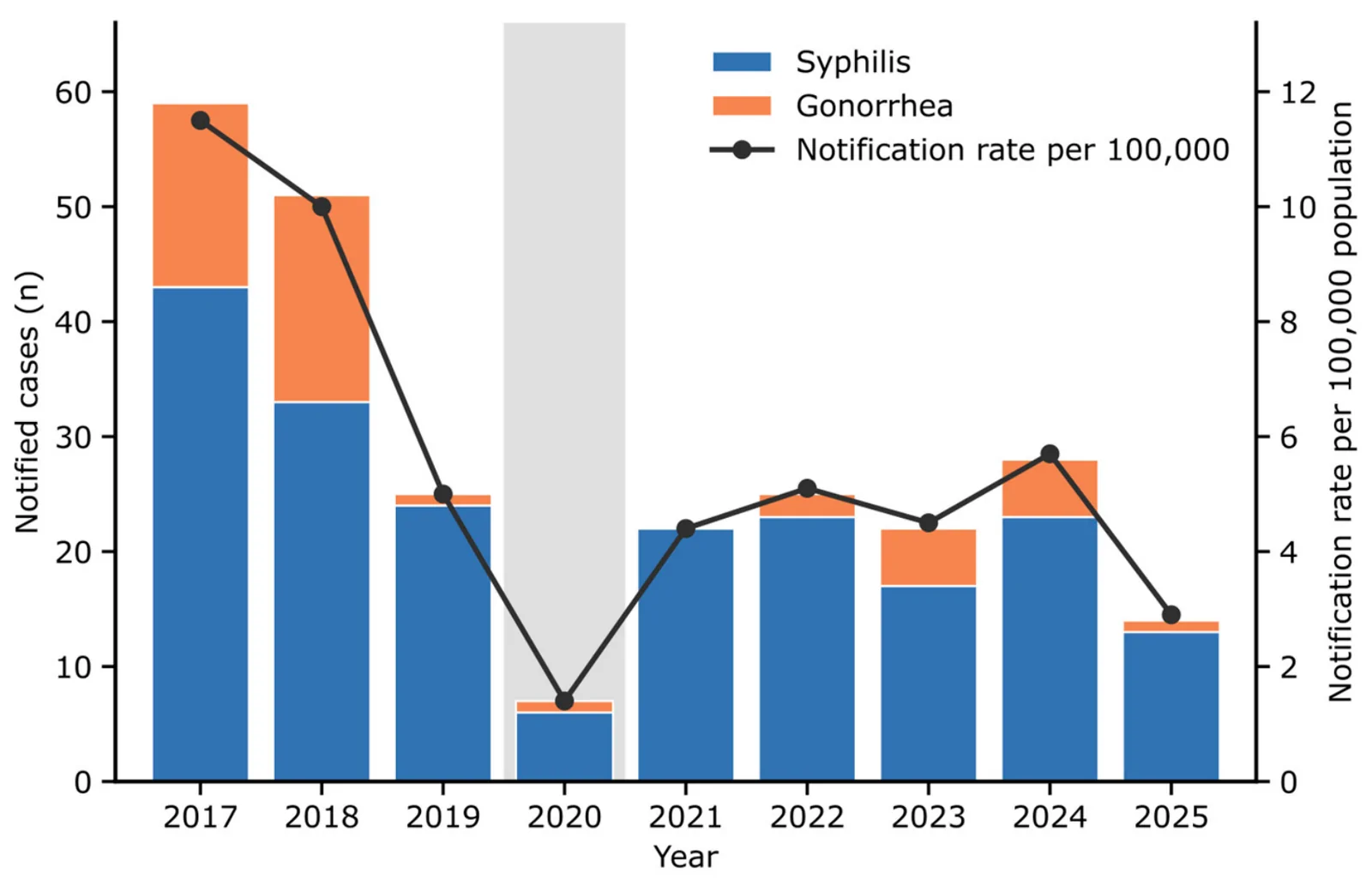
Annual notified syphilis and gonorrhea cases and notification rate, Galați County, 2017–2025. Stacked bars show notified cases (left axis); no chlamydia cases were notified in any year. The line shows the notification rate per 100,000 residents (right axis), derived from the same counts and annual population denominators; it accounts for population change and does not provide independent evidence of the trend. The shaded column marks 2020, the year of COVID-19-related disruption to health services.

Annual case counts ranged from 7 in 2020 to 59 in 2017 (Figure 1). Notification rates declined over the nine years (RR per year = 0.871; 95% CI 0.780–0.973; *p* = 0.014). The decline persisted when 2020 was excluded (RR = 0.870; 95% CI 0.812–0.933; *p* < 0.001) and in a negative binomial model (RR = 0.888; 95% CI 0.801–0.985; *p* = 0.024), indicating that it was not driven solely by the pandemic-year trough. The 2020 nadir (7 cases) coincided with the national COVID-19 state of emergency and the associated disruption of non-urgent outpatient and screening services, providing a plausible structural explanation for that year’s collapse. Notifications partially recovered thereafter, reaching 28 cases in 2024, before falling to 14 in 2025, 42% below the 2021–2024 mean of 24.3 cases per year; given the overdispersion of annual counts, this single-year decrease should be interpreted with caution. An earlier sharp decrease, unrelated to the pandemic, affected gonorrhea, with notifications falling from 18 in 2018 to 1 in 2019; this decrease was concentrated among cases diagnosed in the dermatovenereology service (15 in 2018 versus 1 in 2019). Testing volumes and service activity data were not available to explain either change.

### 3.2. Distribution by Sex and Comparison with the National Level

Over the study period, males accounted for 158 of the 253 cases (62.5%) and females for 95 (37.5%), an overall male-to-female ratio of 1.7:1 (Table 2). This predominance held in most years, with the exception of 2019, when female cases (16) outnumbered male cases (9). Restricted to syphilis alone, the male excess was less marked (114 male versus 90 female cases; ratio 1.3:1), and female cases outnumbered male cases in both 2017 and 2019 (Table 2).

For syphilis specifically, standardized morbidity ratios (SMR) against national reference rates were computed for the 2021–2024 sub-period, for which directly comparable national data were available. The SMR was 2.35 in men (59 observed versus 25.09 expected cases; 95% CI 1.79–3.03; *p* < 0.0001) and 1.76 in women (26 observed versus 14.80 expected cases; 95% CI 1.15–2.57; *p* = 0.011), indicating syphilis notification rates in Galați County significantly higher than expected from national rates in both sexes, with a larger excess among men (Table S1). In 2025, the male-to-female ratio narrowed to 1.3:1 (8 versus 6) from 2.6:1 in 2021–2024 combined; this difference was not statistically significant (Fisher’s exact test, OR = 0.51; *p* = 0.347), and male predominance was maintained throughout, including in 2025.

Gonorrhea showed a stronger male predominance than syphilis: 44 of the 49 cases (89.8%) occurred in men and 5 (10.2%) in women, and no female case was notified in 2018–2022 or in 2024–2025 (Table 3). All 49 gonococcal infections were recorded at a urogenital site; no anorectal or pharyngeal infection was notified. For syphilis, the site of the primary lesion was recorded as genital in all 13 primary-stage cases notified over 2017–2025, whereas the distribution of secondary-stage lesions is not captured by the notification form.

### 3.3. Clinical Stage at Diagnosis

Clinical stage at diagnosis was recorded for 202 of the 204 syphilis cases notified in 2017–2025; stage was unknown for one case in 2018 and one in 2023 (Table S2). Primary-stage disease accounted for 13 cases (6.4%; 95% CI 3.4–10.7%) and secondary-stage disease for 58 (28.4%; 95% CI 22.4–35.2%); 130 cases (63.7%) were latent (65 early latent, 14 late latent and 51 of unspecified duration), and one case was tertiary (Table 4). The distribution across the five principal stage categories differed from a uniform distribution (chi-square = 61.56; df = 4; *p* < 0.0001). Because the timing of infection and of treatment was not recorded, this distribution describes stage at notification and cannot be used to infer diagnostic or treatment delay in individual cases.

### 3.4. Route of Detection and Congenital Syphilis

**Figure 2:**
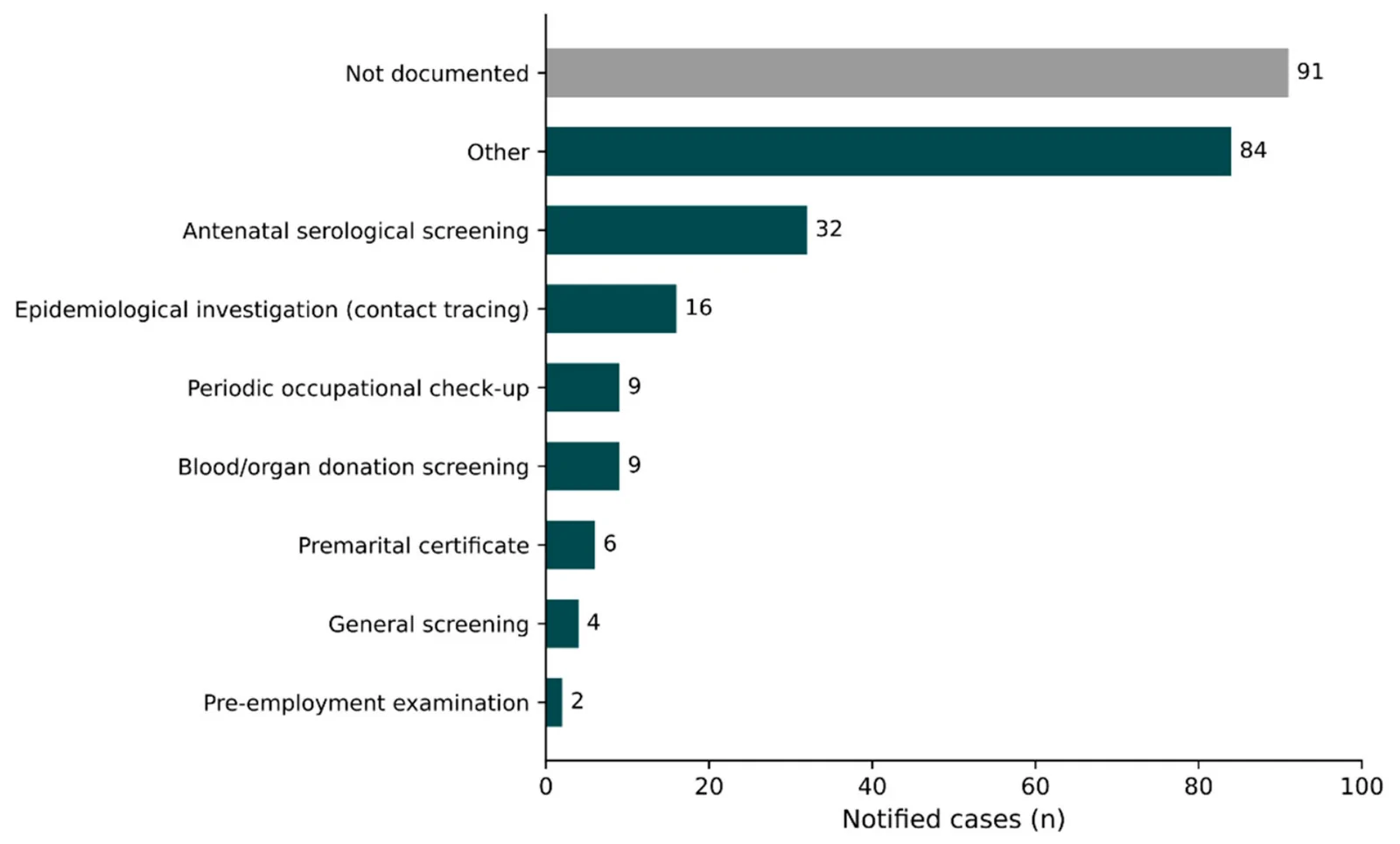
Distribution of notified STI cases by reason for detection, Galați County, 2017–2025.

Across the full 2017–2025 period, the reason for detection was documented for 162 of the 253 cases (64.0%); 91 records (36.0%; 95% CI 30.1–42.2%) lacked this information (Figure 2). Among documented routes, antenatal serological screening was the largest single category (32 cases), followed by epidemiological investigation of contacts (16), periodic occupational check-ups (9), blood or organ donation screening (9), premarital certification (6), general screening (4), and pre-employment examination (2); a further 84 cases were attributed to heterogeneous “other” active case-finding routes not further specified in the source records. This residual category corresponds to the code other on the notification form, for which no further detail is recorded; by place of detection, 54 of these cases were diagnosed in the dermatovenereology service, 27 in other units, 2 in gynecology and 1 in the infectious diseases service. Together with the 91 cases without a recorded reason, 175 of the 253 cases (69.2%) therefore lack a specific detection route, which substantially limits comparisons between defined detection pathways. Despite 32 maternal infections being identified through antenatal screening over the nine-year period, no case of congenital syphilis was notified in Galați County at any point between 2017 and 2025. All 32 maternal infections detected through antenatal screening were syphilis, and none was at the primary stage: 28 were latent (12 early latent, 3 late latent and 13 latent of unspecified duration) and 4 secondary. They were distributed over seven of the nine years (11 in 2017, 4 in 2018, 4 in 2019, 5 in 2021, 2 in 2023, 3 in 2024 and 3 in 2025). The number of pregnant women screened is not recorded in the surveillance database; live births were therefore used as a proxy denominator. Over 2017–2024, the years for which county birth data were available, 29 maternal infections were detected among 38,691 live births in Galați County [16], corresponding to 0.75 per 1000 live births (95% CI 0.50–1.08). The surveillance database records the maternal diagnosis only; maternal treatment, pregnancy outcome and infant serological follow-up are not linked to these records, and congenital cases are notified on a separate form (Section 2.3). The contrast between a structured, mandatory antenatal pathway and a background in which more than a third of all cases carry no documented route of detection illustrates how unevenly the surveillance system performs across its different entry points.

### 3.5. Self-Reported Sexual Behavior

Sexual behavior was recorded for all 253 cases as a single-choice variable with three mutually exclusive categories: heterosexual contact (243 cases, 96.0%; 95% CI 92.9–98.1%), same-sex contact (5 cases), and multiple sexual partners (5 cases). Persons in the last category were therefore not classified by partner sex in this variable; the separate transmission-route field recorded heterosexual transmission for 2 of them, same-sex transmission for 1 and an unknown route for 2. Given the social stigma still attached to disclosure of same-sex contact in this setting, under-reporting cannot be excluded, and this figure should be interpreted as a lower bound on the diversity of behavior actually present.

### 3.6. Co-Infections and HIV Status

For each notified case, the notification form records whether testing for HIV and for other sexually transmitted and blood-borne infections was performed at the time of diagnosis, and its result (Table 5). These data describe testing as documented on the form; clinicians in the main notifying service report that co-testing is performed more often in practice than it is recorded, so the proportions below are likely to underestimate testing actually performed.

HIV testing was documented for 193 of the 253 cases (76.3%), with a marked difference by etiology: 182 of the 204 syphilis cases (89.2%) compared with 11 of the 49 gonorrhea cases (22.4%). Nine cases had a positive HIV result, 7 with syphilis and 2 with gonorrhea, corresponding to 4.7% (95% CI 2.2–8.8%) of the 191 tests with a reported result; one further result was recorded as confidential and one without a result. Seven of the nine HIV-positive cases were men, two of whom reported same-sex contact. The notification form does not indicate whether HIV infection was newly diagnosed or previously known, and use of HIV pre-exposure prophylaxis (PrEP) is not recorded, so neither could be assessed.

Documentation of testing for the other infections was sparse. A chlamydia test result was recorded for 8 cases (3.2%), all negative. Hepatitis B was documented in 2 of 69 tested cases (2.9%), both with syphilis, and hepatitis C in none of 64. Herpes simplex virus infection was recorded in 1 of 9 cases with a documented status, and Mycoplasma genitalium in none of 9 tested. No record documented a concurrent diagnosis of syphilis and gonorrhea.

## 4. Discussion

The present findings indicate that the sexually transmitted infection profile recorded in Galați County over the study period is shaped less by the true transmission of these pathogens than by the intensity and structure of the local case-detection system, a pattern that recurs across every stratum examined below.

The complete absence of *Chlamydia trachomatis* notifications throughout the nine years is the clearest single illustration of this principle. Chlamydia is consistently the most frequently reported bacterial STI across the EU/EEA [15], and its total absence from a county-level dataset of 253 cases is far more consistent with a lack of diagnostic testing than with genuine biological absence. Data from a sexual health clinic in Bucharest illustrate what targeted testing can reveal: in a relatively small and selected, predominantly MSM clinic population, multisite PCR testing detected chlamydia in 8.6% of attendees, while serological testing identified active syphilis in 17.7% [17]. These proportions cannot be extrapolated to the general population of Galați County, but they show that infections absent from notification data can be identified when testing is offered. In Galați, where chlamydia test results were documented for only 8 of the 253 notified patients, although testing is reported to be frequent in clinical practice (Section 2.2 and Section 3.1), the finding is most accurately described as no cases notified in a context in which testing was rarely documented.

The recording of diagnostic practices alongside each case points in the same direction. Chlamydia test results were documented for only 8 of the 253 notified patients, although clinicians in the main notifying service report that patients with syphilis or gonococcal urethritis are frequently tested; the information is therefore lost between clinical care and surveillance, and any positive chlamydia results obtained in these patients did not become notifications. HIV testing was documented for most syphilis cases (89.2%) but for fewer than one in four gonorrhea cases (22.4%), although both infections share transmission routes and gonorrhea facilitates HIV acquisition; these proportions describe documentation on the notification form and may underestimate testing actually performed. All gonococcal infections were recorded at a urogenital site, and no anorectal or pharyngeal infection was notified; where multisite nucleic acid amplification testing is offered, a substantial share of gonococcal infections, particularly among men who have sex with men, is found only at extragenital sites [18]. The nine HIV-positive cases, seven of them with syphilis, are consistent with the syphilis and HIV co-infection pattern described in other Romanian settings [19]. Taken together, these data suggest that co-infections and extragenital infections are poorly captured by the surveillance system, whether because they are not sought or because results are not recorded, and that the documentation of co-testing, not only case counts, should be monitored as a performance indicator of county surveillance.

Annual notification rates declined over the nine years (Section 3.1), a trend that persisted after excluding 2020 and in sensitivity analyses. Even so, the collapse to 7 cases in 2020 coincided with the national COVID-19 state of emergency, during which testing services for HIV, viral hepatitis and STIs were curtailed across the WHO European Region, with the large majority of surveyed providers reporting markedly reduced testing volumes [20]; a comparable disruption in Galați is a plausible explanation for that year’s nadir, although local testing volumes were not available to confirm it. The sharp fall in gonorrhea notifications between 2018 and 2019, which preceded the pandemic and was concentrated in a single service, likewise suggests a change in diagnostic or reporting practice rather than in transmission, but this could not be verified. Superimposed on this longer decline, notifications partially recovered to a local peak of 28 cases in 2024 before falling to 14 in 2025. Syphilis and gonorrhea notifications both decreased in 2025; reduced case-finding activity is one possible explanation, alongside changes in health-care use, reporting completeness or random variation in small annual counts, none of which could be assessed with the available data.

A second consistent feature was a syphilis notification rate that exceeded the national reference rate over 2021–2024 in both sexes, markedly among men (SMR = 2.35; 95% CI 1.79–3.03; *p* < 0.0001) and to a lesser degree among women (SMR = 1.76; 95% CI 1.15–2.57; *p* = 0.011). The excess among women may partly reflect the contribution of mandatory antenatal screening, which accounted for 32 of the 90 syphilis notifications in women over the study period. This asymmetry mirrors the male predominance documented in ECDC syphilis surveillance across the EU/EEA [4,10] and is consistent with evidence from other Romanian settings: a ten-year retrospective series from Sibiu County likewise found syphilis concentrated among urban, working-aged men [21], and a recent single-center cohort of people living with HIV reported syphilis co-infection independently associated with male sex, urban residence and unmarried status [19]. The Galați data do not allow the male excess to be attributed to specific subgroups: same-sex contact was reported for only 5 of the 114 male syphilis cases, and under-reporting of same-sex contact is likely (Section 3.5). A contribution from men who have sex with men, as described in European surveillance and in other Romanian settings [4,19], is therefore a possible explanation rather than a finding of this study.

The most internally consistent evidence for the case-detection hypothesis comes from the contrast between antenatal and non-antenatal detection. Structured, mandatory serological screening in pregnancy identified 32 maternal syphilis infections over the nine years, the single largest documented detection route, yet not one case of congenital syphilis was notified in Galați County throughout 2017–2025. Because pregnancy outcomes and infant follow-up were not linked to the maternal records, the absence of congenital syphilis notifications cannot by itself demonstrate that transmission was prevented in each of these pregnancies; it is, however, consistent with WHO guidance identifying early screening and treatment in pregnancy as the central, highly cost-effective intervention for eliminating congenital syphilis [22], and with global estimates attributing a substantial share of the worldwide congenital syphilis burden to gaps in antenatal coverage rather than treatment failure [23]. This absence of notified congenital cases contrasts with the wider trajectory, in which ECDC data show congenital syphilis notifications climbing sharply since 2021 [24], and US surveillance has similarly attributed most congenital cases to missed or delayed antenatal screening rather than to treatment failure once infection is identified [25]. The antenatal circuit is the one segment of local surveillance that is structured and mandatory. Its yield was not constant over time, however, ranging from 11 maternal infections in 2017 to none in 2020 and 2022, and without annual numbers of women screened it is not possible to determine whether this variation reflects screening coverage or prevalence. As with chlamydia, the absence of congenital syphilis cases means only that none was notified; neither finding, taken alone, measures true occurrence. The two situations differ, however, in the testing context: chlamydia testing was rarely documented and not systematic, whereas pregnant women were covered by a mandatory screening pathway, so that the absence of congenital notifications occurred where maternal detection was structured.

Read together, these patterns (the etiological gap left by chlamydia, the pandemic-linked and post-pandemic fluctuations, the excess over national rates, larger among men, and the contrast between antenatal and opportunistic detection) are more consistent with a notification profile shaped by the structure of case detection than with an unusually high or rapidly changing burden of sexual transmission, although, in the absence of testing and outcome data, this interpretation remains a hypothesis. This reading is consistent with the longer Romanian literature, which has repeatedly described national STI notification data as more reflective of testing infrastructure than of underlying transmission [26], and with the observation that self-reported behavioral data of the kind collected here (96.0% heterosexual transmission among the 253 cases with recorded behavior) tend to understate the diversity of sexual behavior where disclosure of same-sex contact carries social stigma [19,27].

Several limitations should be considered. Annual caseloads were small (7–59 cases per year), limiting statistical power for subgroup and year-specific comparisons; pooling several years improves the precision of the estimates reported here but does not reduce potential systematic bias. The route of detection was undocumented for over a third of cases (36.0%; 95% CI 30.1–42.2%), which may bias the apparent distribution of detection pathways. Behavioral variables were self-reported and likely subject to under-disclosure, particularly for same-sex contact. The national comparator used for standardized morbidity ratios was available only for 2021–2024, so no equivalent county-versus-national comparison could be made for 2025 or the earlier study years. The data contain no population testing denominators or positivity rates, so the number of persons tested for syphilis, gonorrhea or chlamydia, including the number of pregnant women screened, is unknown; no treatment information or linked maternal-infant outcomes were available; and no formal assessment of case-ascertainment completeness could be performed. Consequently, the study cannot distinguish changes in transmission from changes in testing, diagnosis or reporting, and the interpretations favoring case detection are hypotheses consistent with, but not demonstrated by, the data. Co-testing results were available only for notified cases, and tests performed in clinical practice, including chlamydia tests, were not systematically recorded on the notification form; PrEP use and the timing of HIV diagnosis were not recorded, and 69.2% of cases lacked a specific detection route. Finally, contact-tracing and partner-notification data, although collected in the surveillance database, were absent for 2023 and incomplete in other years, precluding a systematic analysis of partner-notification performance.

## 5. Conclusions

Over the nine years examined, notifications in Galați County consisted of syphilis and, in smaller numbers, gonorrhea; no chlamydia case was notified, in a context in which chlamydia testing was rarely documented. Notification rates declined over the period, with a trough in 2020 and partial recovery thereafter, a trajectory compatible with variation in testing and reporting activity, although the available data cannot separate this from changes in transmission. Syphilis notification rates exceeded those expected from national rates in both sexes, with a larger excess among men.

Three practical priorities follow for county-level public health. The first is earlier diagnosis: only 6.4% of staged syphilis cases were notified at the primary stage. The second is the evaluation of partner notification, which could not be assessed because the corresponding records were incomplete. The third is diagnostic capacity for gonorrhea and chlamydia, including extragenital sampling and routine co-testing, without which the local etiological profile will continue to describe what is tested rather than what circulates.

Antenatal screening, the only structured and mandatory pathway in this system, produced the largest group of cases with a documented detection route, and no congenital syphilis case was notified in the county between 2017 and 2025. Whether structured testing pathways detect infections more effectively than opportunistic ones could not be tested directly and should be examined in studies that include testing denominators and outcome data.

## Figures and Tables

**Table 1:** Annual distribution of notified sexually transmitted infection cases by etiology, Galați County, 2017–2025.

Year	Syphilis (*n*)	Gonorrhea (*n*)	Chlamydia (*n*)	Total (*n*)	Population	Notification Rate per 100,000
2017	43	16	0	59	515,282	11.5
2018	33	18	0	51	510,865	10.0
2019	24	1	0	25	504,058	5.0
2020	6	1	0	7	500,213	1.4
2021	22	0	0	22	495,149	4.4
2022	23	2	0	25	495,030	5.1
2023	17	5	0	22	494,280	4.5
2024	23	5	0	28	493,009	5.7
2025	13	1	0	14	489,056	2.9
**Total**	**204**	**49**	**0**	**253**	**—**	**5.6**

Notification rates were calculated using the resident population of Galați County on 1 January of each year, as published by Eurostat for NUTS 3 region RO224 [13]; values from 2020 onwards are Eurostat estimates following the 2021 census revision. The Total row reports the mean annual notification rate over the nine years. Quasi-Poisson regression of annual notifications on calendar year with a population offset: RR per year = 0.871 (95% CI 0.780–0.973), *p* = 0.014.

**Table 2:** Distribution of notified cases by sex, for all sexually transmitted infections and for syphilis separately, Galați County, 2017–2025, with syphilis-specific standardized morbidity ratios (SMR) against the national reference, 2021–2024.

Year	All Notified STI Cases			Syphilis Cases Only		
	Male (*n*)	Female (*n*)	M:F Ratio	Male (*n*)	Female (*n*)	M:F Ratio
2017	30	29	1.03	18	25	0.72
2018	36	15	2.40	18	15	1.20
2019	9	16	0.56	8	16	0.50
2020	5	2	2.50	4	2	2.00
2021	14	8	1.75	14	8	1.75
2022	19	6	3.17	17	6	2.83
2023	16	6	2.67	12	5	2.40
2024	21	7	3.00	16	7	2.29
2025	8	6	1.33	7	6	1.17
**Total**	**158**	**95**	**1.66**	**114**	**90**	**1.27**

Syphilis-specific SMR, 2021–2024 vs. national reference: male 2.35 (95% CI 1.79–3.03), *p* < 0.0001; female 1.76 (95% CI 1.15–2.57), *p* = 0.011. Expected counts are based on the usual resident county population (Eurostat). Observed and expected counts by year are given in Table S1. National comparator not available for 2025.

**Table 3:** Notified gonorrhea cases by year, sex, anatomical site of infection and documented HIV testing, Galați County, 2017–2025.

Year	Male *n*	Female *n*	Total *n*	Urogenital Site *n*	Extragenital Site *n*	HIV Test Documented *n*
2017	12	4	16	16	0	3
2018	18	0	18	18	0	1
2019	1	0	1	1	0	0
2020	1	0	1	1	0	0
2021	0	0	0	0	0	0
2022	2	0	2	2	0	0
2023	4	1	5	5	0	4
2024	5	0	5	5	0	2
2025	1	0	1	1	0	1
Total	44 (89.8)	5 (10.2)	49	49 (100.0)	0 (0.0)	11 (22.4)

Values in the Total row are n (%) of the 49 gonorrhea cases. Anatomical site as recorded on the notification form. HIV testing refers to a test result documented on the notification form (Section 3.6).

**Table 4:** Distribution of syphilis cases by clinical stage at diagnosis, Galați County, 2017–2025.

Clinical Stage	*n*	%	95% CI
Primary	13	6.4	3.4–10.7
Secondary	58	28.4	22.4–35.2
Early latent	65	31.9	25.5–38.7
Late latent	14	6.9	3.8–11.2
Latent, unspecified	51	25.0	19.2–31.5
Tertiary	1	0.5	—
Unknown	2	1.0	—
Total	204	100.0	

Chi-square = 61.56, df = 4, *p* < 0.0001 (five principal categories). Clopper–Pearson 95% CI. Stage was unknown for 2 of the 204 cases.

**Table 5:** Documented testing for HIV and other sexually transmitted and blood-borne infections at the time of diagnosis, by etiology, among notified syphilis and gonorrhea cases, Galați County, 2017–2025.

Infection Tested at Diagnosis	Syphilis (*n* = 204) Documented, *n* (%)	Positive, *n*	Gonorrhea (*n* = 49) Documented, *n* (%)	Positive, *n*	All Cases (*n* = 253) Documented, *n* (%)	Positive, *n*
HIV	182 (89.2)	7	11 (22.4)	2	193 (76.3)	9
Chlamydia trachomatis	7 (3.4)	0	1 (2.0)	0	8 (3.2)	0
Hepatitis B	64 (31.4)	2	5 (10.2)	0	69 (27.3)	2
Hepatitis C	59 (28.9)	0	5 (10.2)	0	64 (25.3)	0
Herpes simplex virus	8 (3.9)	1	1 (2.0)	0	9 (3.6)	1
Mycoplasma genitalium	8 (3.9)	0	1 (2.0)	0	9 (3.6)	0

Documented: a test result recorded on the notification form; percentages refer to all cases in the column. Among all cases, positive results represent 4.7% of the 191 HIV tests with a reported result (one recorded as confidential and one without a result), 2.9% of hepatitis B tests and 11.1% of cases with a recorded herpes simplex virus status. No concurrent syphilis and gonorrhea diagnosis was recorded, and use of HIV pre-exposure prophylaxis is not recorded on the notification form.

## Data Availability

The data presented in this study are not publicly available. They were extracted from the surveillance database maintained by the Galați County Public Health Directorate (DSP Galați) and were released to the authors, in de-identified form, following a request submitted under Law No. 544/2001 on free access to information of public interest. Access to the underlying case-level records therefore rests with DSP Galați rather than with the authors, and researchers wishing to use the source data would need to submit an equivalent request to that institution. Summary data supporting the results reported here can be made available by the corresponding author upon reasonable request.

## Supplementary Materials

The following supporting information can be downloaded at: https://www.mdpi.com/article/10.3390/diseases14100373/s1, Table S1: Sex-specific syphilis standardized morbidity ratios (SMR), Galați County versus Romania, 2021–2024: national reference rates, county populations, and observed and expected cases; Table S2: Clinical stage at diagnosis of notified syphilis cases by year, Galați County, 2017–2025.

## Abbreviations

The following abbreviations are used in this manuscript:

CI	Confidence interval
CNSCBT	National Centre for Surveillance and Control of Communicable Diseases
DSP	County Public Health Directorate (Direcția de Sănătate Publică)
ECDC	European Centre for Disease Prevention and Control
EU/EEA	European Union/European Economic Area
GDPR	General Data Protection Regulation
INSP	National Institute of Public Health
RR	Rate ratio
MSM	Men who have sex with men
SMR	Standardized morbidity ratio
STI	Sexually transmitted infection
