# Supplementary material for: Syphilis, Gonorrhea, and Chlamydia Notifications in Galați County, Romania (2017–2025): A Retrospective Surveillance Analysis
Source: Diseases. 2026 Oct 11;14(10):373. doi: 10.3390/diseases14100373 (PMC13702969; doi:10.3390/diseases14100373)
Supplement: Supplementary file 1 [file diseases-14-00373-s001.zip › diseases-4554632-supplementary.pdf]

Supplementary Materials

Syphilis, Gonorrhea, and Chlamydia Notifications in Galați County, Romania (2017–2025): A Retrospective Surveillance Analysis

Ionelia State, Laura Cristescu-Budală, Rares-Nicolae Vadana, Alin-Laurentiu Tatu, Laurentiu-Tony Hangan and Simona-Dana Mitincu-Caramfil

Table S1. Sex-specific syphilis standardized morbidity ratios (SMR), Galați County versus Romania, 2021–2024: national reference rates, county populations, and observed and expected cases.

Sex	Year	National rate per 100,000	Galați population	Expected (E)	Observed (O)	SMR (95% CI)	p
Men	2021	1.87	243,294	4.55	14	3.08 (1.68–5.16)	< 0.001
	2022	3.20	241,053	7.71	17	2.20 (1.28–3.53)	0.005
	2023	1.68	240,844	4.05	12	2.97 (1.53–5.18)	0.002
	2024	3.65	240,514	8.78	16	1.82 (1.04–2.96)	0.036
	**2021–2024**	—	—	**25.09**	**59**	**2.35 (1.79–3.03)**	**< 0.0001**
Women	2021	1.03	251,855	2.59	8	3.08 (1.33–6.08)	0.011
	2022	1.41	253,977	3.58	6	1.68 (0.61–3.65)	0.307
	2023	1.76	253,436	4.46	5	1.12 (0.36–2.62)	0.921
	2024	1.65	252,495	4.17	7	1.68 (0.68–3.46)	0.257
	**2021–2024**	—	—	**14.80**	**26**	**1.76 (1.15–2.57)**	**0.011**

National rates: sex-specific syphilis notification rates for Romania, from the annual reports of the National Institute of Public Health, National Centre for Surveillance and Control of Communicable Diseases (INSP–CNSCBT) [1]. Galați population: usually resident population on 1 January, Eurostat, NUTS 3 region RO224 [2], the same denominators used for the notification rates in Table 1 of the main text. Expected cases (E) = national rate × Galați population / 100,000. SMR = O/E. 95% CI: exact Poisson confidence interval for the observed count (chi-square method). p: two-sided exact Poisson test of O against E (scipy.stats.poisson). Annual SMRs are shown for completeness; given the small annual counts, inference is based on the pooled 2021–2024 estimates. National data for 2025 were not available.

Table S2. Clinical stage at diagnosis of notified syphilis cases by year, Galați County, 2017–2025.

Year	Primary	Secondary	Early latent	Late latent	Latent, unspecified	Tertiary	Unknown	Total
2017	3	7	19	10	4	0	0	43
2018	1	5	15	2	8	1	1	33
2019	1	1	9	0	13	0	0	24
2020	1	3	0	0	2	0	0	6
2021	0	12	3	0	7	0	0	22
2022	1	11	4	0	7	0	0	23
2023	0	8	5	1	2	0	1	17
2024	3	9	5	0	6	0	0	23
2025	3	2	5	1	2	0	0	13
**Total, n (%)**	**13 (6.4)**	**58 (28.4)**	**65 (31.9)**	**14 (6.9)**	**51 (25.0)**	**1 (0.5)**	**2 (1.0)**	**204 (100.0)**

Values are numbers of cases; the Total row shows n (%) of the 204 syphilis cases. Stage as assigned by the diagnosing dermatovenereologist and recorded on the notification form, according to Ministry of Health Order No. 1342/2013. Unknown: stage not recorded. No congenital syphilis case is recorded in this database; congenital cases are notified on a separate form (main text, Section 2.3).

References

1. Analiza bolilor transmisibile aflate în supraveghere – Raport pentru anul 2024; Institutul Național de Sănătate Publică: București, Romania, 2025.

2. Eurostat. Population on 1 January by Broad Age Group, Sex and NUTS 3 Region (demo_r_pjanaggr3). Available online: https://ec.europa.eu/eurostat/databrowser/view/demo_r_pjanaggr3 (accessed on 23 September 2026).
